# Exploring the Influence of Environmental Factors on Bacterial Communities within the Rhizosphere of the Cu-tolerant plant, *Elsholtzia splendens*

**DOI:** 10.1038/srep36302

**Published:** 2016-10-26

**Authors:** Longfei Jiang, Mengke Song, Li Yang, Dayi Zhang, Yingtao Sun, Zhenguo Shen, Chunling Luo, Gan Zhang

**Affiliations:** 1 College of Life Sciences, Nanjing Agricultural University, Nanjing 210095, China; 2 Guangzhou Institute of Geochemistry, Chinese Academy of Sciences, Guangzhou 510640, China; 3 Lancaster Environment Centre, Lancaster University, Lancaster, LA1 4YW, UK

## Abstract

Bacterial communities of rhizospheric soils play an important role in the tolerance and uptake of metal-tolerant/hyperaccumulating plants to metals, e.g. the Cu-tolerant *Elsholtzia splendens* native to China. In this work, pyrosequencing of the bacterial 16S rRNA gene was firstly applied to investigate the rhizospheric bacterial community of *E. splendens* grown at Cu contaminated sites. The 47 phyla including 11 dominant phyla (>1%) in *E. splendens* rhizosphere were presented. The effects of Cu and other environmental factors (total organic carbon, total nitrogen and pH) on the rhizospheric bacterial community were studied comprehensively. The phyla abundances were affected by the environmental factors to different extent, and we found pH, instead of Cu concentration, influenced UniFrac distance significantly and was identified as the most important environmental factor affecting bacterial community. In addition, the influence of environmental factors on gene profiles was explored according to the predicted metagenomes obtained by PICRUSt (phylogenetic investigation of communities by reconstruction of unobserved states). Our study illustrates a view about Cu-tolerant *E. splendens* rhizospheric bacterial communities (composition, diversity and gene profiles) and their influencing factors, giving a hand for the understanding on bacterial community is formed and affected in rhizosphere.

Soil contamination by metals has been of a great concern on a global scale because of their threats to the environment, food security and human health. Cu is one of these metals, although it is an essential micronutrient for normal plant growth and development, which can impair and damage plant growth when exceeding the optimal concentration^1^,^2^. Various physical and chemical technologies have been developed to remediate soils contaminated with metals, and phytoremediation is becoming increasingly popular because of its economic, energy-efficient, and environmentally friendly features^3^. *Elsholtzia splendens*, native to China, has gained special attention due to its high tolerance to Cu^4^. Studies have revealed the capability of *E. splendens* to enhance metal solubility via a root-bacteria interaction, absorb metals with metal transporters, and detoxify metals by chelating and distributing them to the apoplast^5^,^6^,^7^.

Rhizospheric bacteria are crucial for the success of phytoremediation. Normally, the low solubility and availability of metals in most soils has limited the performance of phytoremediation. Rhizospheric bacteria can improve phytoremediation through altering metal bioavailability by changing the environmental pH, producing chelating substances and altering redox potentials^8^. Additionally, bacteria can increase metal removal efficiency via promoting plant growth by synthetizing plant hormones, secreting enzymes, producing siderophores and solubilizing phosphate in soils^9^. Research on *E. splendens* rhizospheric bacteria reveals that Cu-tolerant bacteria help in enhancing soil Cu solubility in *E. splendens* rhizosphere, and inoculating roots with bacteria almost doubles the shoot Cu concentration^10^,^11^. Therefore, bacterial communities within rhizosphere, especially the metal-tolerant plants such as *E. splendens*, become attractive and numerous researches attempt to elucidate their influencing factors.

Metals can alter bacterial abundance, community structure and diversity^12^,^13^. Smit *et al*. found that Cu affects the bacterial composition and reduces the microbial diversity severely by comparing the patterns of Amplified Ribosomal DNA Restriction Analysis (ARDRA) between clean and contaminated soils^14^. Though recovered to some extent attributing to microbial adaptation, the alteration of bacterial community composition and declining diversity are linked to metals in long-term contaminated soils^15^,^16^,^17^. The laboratory-based work suggested different results led by the metal-tolerant plant rhizospheric environment that *E. splendens* has higher capability than the non-Cu-accumulator *Trifolium repens* to maintain rhizospheric bacterial activities and composition^18^. Nevertheless, the laboratory investigation involves the conditions differing considerably from real situations in the field^19^. Besides, though several Cu-tolerate bacteria have been isolated from *E. splendens* rhizosphere, such as *Actinobacteria, Firmicutes* and *Proteobacteria*, the relationship between Cu and rhizospheric microbes still remains unclear^18^,^20^. To date, few studies give a comprehensive view regarding the influence of Cu on the composition, diversity and gene abundance of the rhizospheric bacterial community associated with *E. splendens* growing *in situ* at Cu mine sites.

Other environmental factors in soils, such as total organic carbon (TOC), total nitrogen (TN) and pH, also affect indigenous bacterial community profiles. For example, pH explains the varying diversity and richness of soil bacterial communities in two ecosystems across North and South America (*r*^2^ = 0.70 and *r*^2^ = 0.58)^21^; carbon resources and nitrogen depositions alter bacterial communities and form diversity patterns^22^,^23^,^24^. However, limited information about the roles of these individual environmental factors on *E. splendens* rhizospheric bacterial community is available at Cu mine sites, although their impacts on bacterial community derived from a hyperaccumulating plant rhizosphere have been investigated in several studies^25^,^26^,^27^.

In this study, we sampled 21 soils with different Cu concentrations from three provinces in China with the aim to illustrate a comprehensive picture about *E. splendens* rhizospheric bacterial communities including composition, diversity and gene profiles, and to disclose which environmental factor is the most important one influencing rhizospheric bacterial community. Tag pyrosequencing of the V4 region of the 16S rRNA gene was performed to investigate bacterial community composition and provided much more detailed insights. The metagenomic content and abundance of gene families in 21 samples were predicted using PICRUSt method^28^. These data allow our more comprehensive understanding on not only the gene profiles in rhizospheric soils but also the relationship between them and environmental factors. According to our knowledge, this is the first time investigating the bacterial community within *E. splendens* rhizosphere *in situ* at Cu mine sites, which extends our horizons on the rhizospheric bacteria of metal-tolerant plants.

## Results

### Soil physicochemical characteristics

The soil samples covered a wide range of both total and extractable Cu concentrations, ranging from 728 ± 24 (Mean ± S.D.) to 5,300 ± 430 mg kg^−1^ and 5.19 ± 0.13 to 92.1 ± 1.03 mg kg^−1^, respectively (Supplementary Table S1). All soil samples were of low fertility with TOC < 8.80‰ and TN < 1.17‰ (Supplementary Table S1). The pH values varied from 3.7 to 6.8 with huge differences. No significant relationship between pH and extractable Cu concentrations (*p* = 0.936) was discovered.

### Composition and diversity of rhizospheric bacterial communities

**Figure 1:**
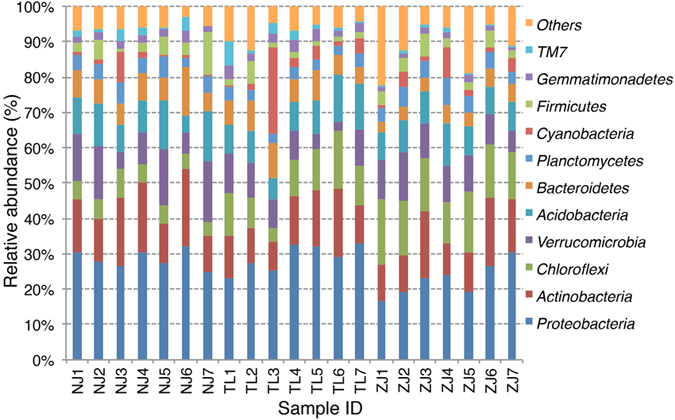
Relative abundances (%) of 11 dominant bacterial phyla across all soil samples. Others composed of 36 rare phyla (<1%) and unclassified bacteria.

After removing low-quality sequences, 1,706,691 sequences were generated from 21 samples, with an average of 81,271 ± 15,318 (Mean ± S.D.) and a range from 51,403 to 106,985 sequences per sample. Based on the classifiable sequences, 47 phyla of bacteria were identified. The major lineages (>1%) of total sequences were in the following order: *Proteobacteria* (26.7%) > *Actinobacteria* (13.9%) > *Chloroflexi* (10.3%)> *Verrucomicrobia* (9.80%)*> Acidobacteria* (9.52%)> *Bacteroidetes* (6.43%)> *Planctomycetes* (4.28%)> *Cyanobacteria* (3.54%)> *Firmicutes* (3.53%)> *Gemmatimonadetes* (2.17%)> *TM7* (1.60%) (Fig. 1). Moreover, 5.04% of sequences were classified as unknown phyla affiliated with bacteria. The rare phyla (abundance <1%) contained <3.09% of the total sequences.

When grouped at a 97% similarity level, 34,214 OTUs were observed across all the samples with a range from 5,792 to 9,144 OTUs per sample. The α-diversity indices (Chao1, Shannon and Simpson) and the observed species were calculated and listed in Table 1. The Chao1, Shannon index and observed species ranged from 8,561 to 12,175, 8.79 to 10.71, and 5,792 to 9,144, respectively. Owing to the high depth of Illumina sequencing, the Simpson index had an average value of 0.9926, nearly 1. To estimate the sequencing coverage, rarefaction curves were plotted (Supplementary Figure S1) based on observed OTUs. The β-diversity (weighted Unifrac distance), representing the bacterial community similarity among samples, was shown in the supplementary (Supplementary Figure S2).

### Relationship between environmental factors and bacterial community composition and diversity

**Figure 2:**
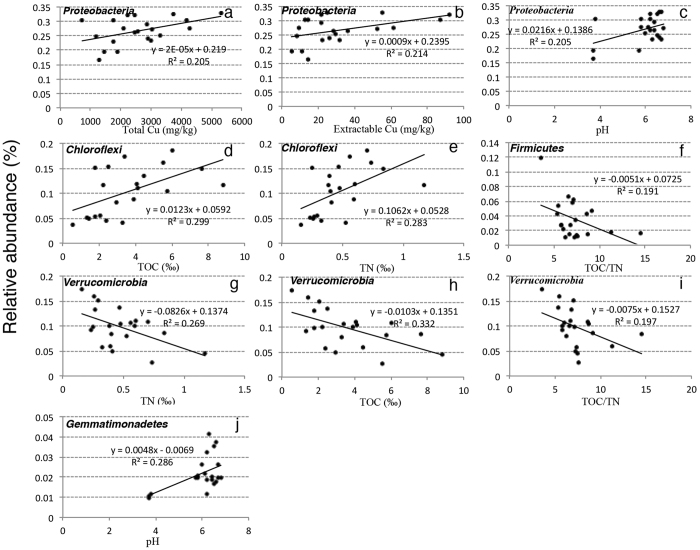
Relationship between five major lineages phyla abundance and environmental factors (*p* < 0.05): (**a**) *Proteobacteria* against total Cu, (**b**) *Proteobacteria* against extractable Cu, (**c**) *Proteobacteria* against pH, (**d**) *Chloroflexi* against TOC, (**e**) *Chloroflexi* against TN, (**f**) *Firmicutes* against TOC/TN, (**g**) *Verrucomicrobia* against TN, (**h**) *Verrucomicrobia* against TOC, (**i**) *Verrucomicrobia* against TOC/TN, and (**j**) *Gemmatimonadetes* against pH.

Five of the eleven major phyla (>1%) were linearly correlated with Cu or other individual environmental factors (*p* < 0.05). *Proteobacteria*, the most abundant phylum across all soil samples, showed significantly positive correlations with total Cu (Fig. 2a, *r*^*2*^ = 0.205, *p* = 0.039), extractable Cu (Fig. 2b, *r*^*2*^ = 0.214, *p* = 0.035) and pH (Fig. 2c, *r*^2^ = 0.205, *p* = 0.039). The relative abundance of *Proteobacteria* increased with the Cu concentration and pH value. *Chloroflexi* had a positive relationship with TOC (Fig. 2d, *r*^2^ = 0.299, *p* = 0.010) and TN (Fig. 2e, *r*^2^ = 0.283, *p* = 0.013), indicating its sensitivity to nutrition. *Firmicutes* was negatively correlated with TOC/TN (Fig. 2f, *r*^2^ = 0.192, *p* = 0.047), and *Verrucomicrobia* showed a negative correlation with TN (Fig. 2g, *r* = 0.269, *p* = 0.016), TOC (Fig. 2h, *r*^*2*^ = 0.332, *p* = 0.006) and TOC/TN (Fig. 2i, *r*^2^ = 0.197, *p* = 0.044), respectively. Besides, pH influenced *Gemmatimonadetes* significantly (Fig. 2j, *r*^2^ = 0.286, *p* = 0.012). No significant impact was identified when analyzing the relationships between the OTUs in these five phyla and environmental factors based on canonical correspondence analysis (CCA).

**Figure 3:**
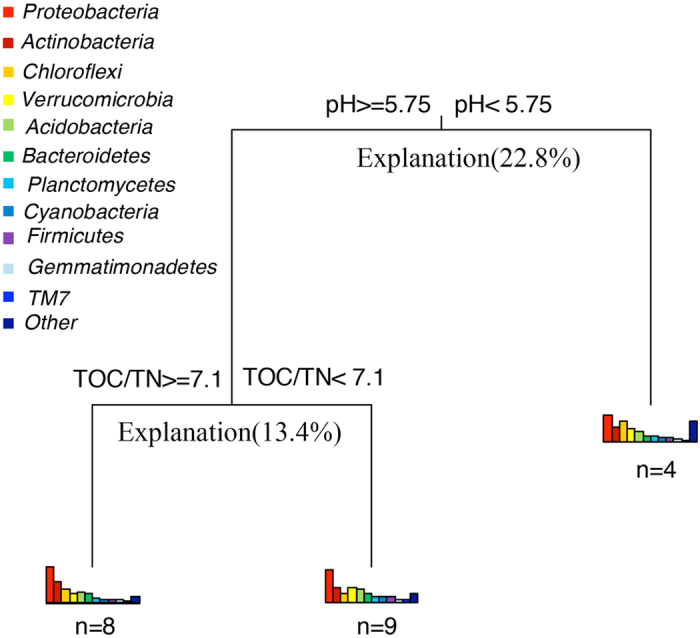
Multivariate regression tree (MRT) analysis of the relation between relative abundance of 11 dominant phyla and environmental factors. Bars plotted under each cluster represent the relative abundance of each phylum. The distribution patterns of relative abundance represent the dynamics of community composition among each split. The numbers under the bars are the number of samples in each group.

**Figure 4:**
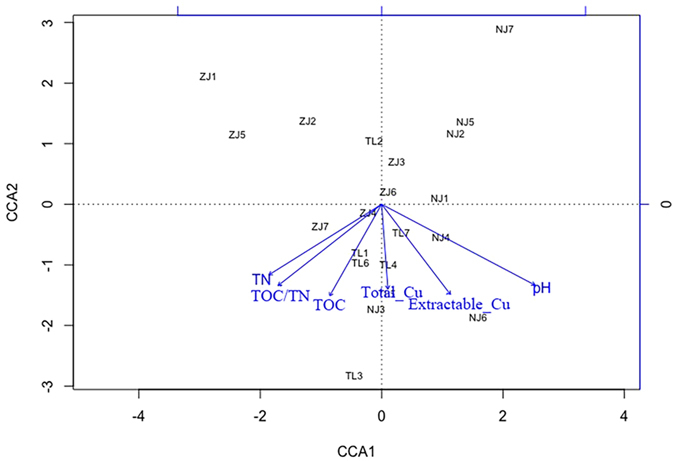
Canonical correspondence analysis (CCA) of bacterial community composition and environmental factors. TOC/TN and pH are significantly related to bacterial community composition.

All the tested environmental factors clearly affected the bacterial community composition and multivariate regression tree (MRT) was used to reveal the most influential factor. The results suggested that pH was the most sensitive predictor of the relative abundance (Fig. 3), consistent with the conclusion from our Pearson’s correlation analysis that two phyla, *Proteobacteria* (the most abundance phylum) and *Gemmatimonadetes*, were strongly affected by pH. The ratio of TOC/TN, relating to *Verrucomicrobia* and *Firmicutes*, was identified as the second major environmental factor and showed a powerful influence on bacterial community composition. The average phyla abundances of each split in MRT nodes were presented in Table 2 and their variance explanation was exhibited in Fig. 3. Parallelly, the results of CCA showed that pH (*p* = 0.004) and TOC/TN (*p* = 0.040) explained the bacterial community most (Fig. 4).

The relationship between bacterial α-diversity and environmental factors was interpreted in Supplementary Table S2. No obvious correlation was detected except for the observed species negatively correlated with TOC/TN. The Mantel test by assessing the correlation between UniFrac distance and environmental factors distance matrix, which was used in this study to estimate the phylogenetic distance affected by environmental factors, indicated that pH value was the most important factor in bacterial evolution (*r*^2^ = 0.246, *p* = 0.005). Meanwhile, no other environmental factor was related to the bacterial evolutionary distance.

### Metagenome prediction and the relationship between environmental factors and functional gene profiles

**Figure 5:**
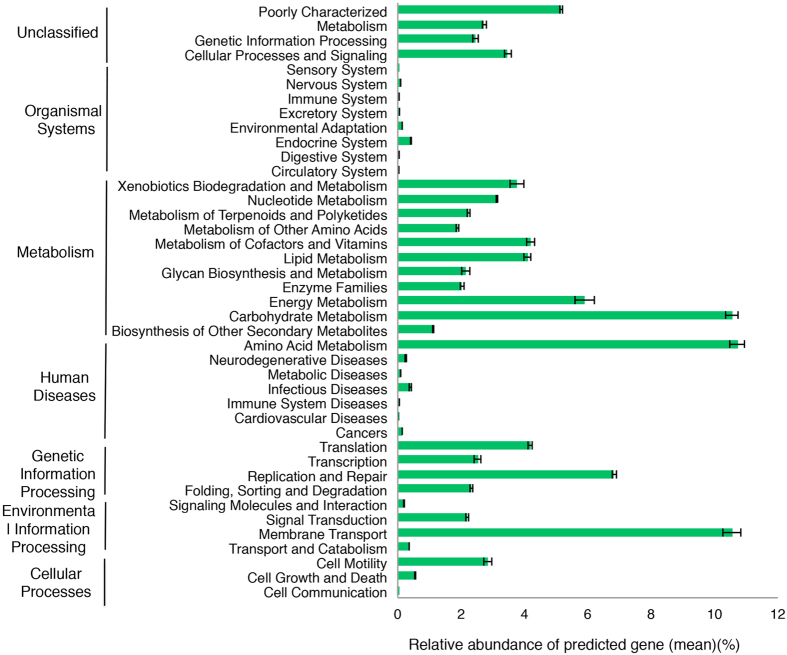
Gene profiles of bacterial community in *E. splendens* rhizosphere predicted using PICRUSt.

PICRUSt approach was applied to infer the metagenomic content of the samples, and to evaluate the functional potential of the bacterial community’s metagenome from its 16S profile. Although this method is limited by the number of available genomes, it has been shown to replicate metagenomes to a high degree of accuracy^28^. The Nearest Sequenced Taxon Index (NSTI) quantified the availability of nearby genome representatives for each sample, and its value was 0.17 ± 0.02 (Mean ± S.D.) in Langille’s study. Our work showed a similar NSTI value (0.17 ± 0.01, Supplementary Table S3), indicating that enough and closely related reference genomes were available for the dataset. Based on the predicted metagenomes, 41 of level 2 KEGG Orthology (KO) groups were found and the gene families belonging to amino acid metabolism, carbohydrate metabolism and membrane transport were identified as the major gene families (Fig. 5). To elucidate the relationship between environmental factors and gene families, the Pearson’s correlation was performed. Total Cu was negatively related with carbohydrate (*p* = 0.042) and lipid (*p* = 0.012) metabolism gene abundance. With the decreasing pH value, the gene abundances of “biosynthesis of secondary metabolites” (*p* = 0.010), “signaling molecules and interaction” (*p* = 0.001) and “transcription” (*p* = 0.019) increased significantly.

## Discussion

In this study, we presented the composition of bacterial community in *E. splendens* rhizosphere at Cu mine sites. The community composition (Fig. 1) provided much deeper information *in situ* comparing with previous studies. Although the laboratory research gave us some insight in bacterial composition in *E. splendens* rhizosphere^18^, no more than 19 bacterial species were identified due to the limited resolution of denatured gradient gel electrophoresis (DGGE). Here, with the help of pyrosequencing, 47 phyla were found and the abundance of 11 phyla was larger than 1%. The results indicated that the bacterial communities in *E. splendens* rhizosphere are quite diversified and more complicated than we detected before^18^.

To understand the relationship between environmental factors and bacterial community in *E. splendens* rhizosphere, the Pearson’s correlation analysis illustrated the increasing abundance of *Proteobacteria* with the Cu concentration, showing the capability of the bacteria affiliated to this phylum to tolerate Cu contamination in rhizosphere. This tolerance may be attributed to an array of metal transport systems in *Proteobacteria*, as reported in a deep-sea genome analysis^29^. In addition, several isolated integrons and plasmids are found to be broadly disseminated among *Proteobacteria*^30^,^31^, which allows the resistance genes transfer from one bacterium to another. Such horizontal gene transfer mechanisms might explain the increasing abundance of *Proteobacteria* with Cu pressure.

Surprisingly, the present study found weak influence of Cu on rhizospheric bacterial community, different from previous studies. Here, MRT and CCA illustrated limited effects of Cu on the change of bacterial community composition (Figs 3 and 4). There was also no relationship discovered between Cu concentration and bacterial diversity, including α- and β-diversity. Additionally, the rarefaction curve (Supplementary Figure S1) also revealed the high diversity level, which suggested that Cu does not have significant impacts on rhizospheric bacterial community diversity. While, previous researches reported that metals can influence the bacterial community composition greatly^32^. High concentrations of metals decrease the Chao1^33^ and Shannon index^34^, and alter the Simpson index^35^. Comparing to these studies, the unique feature of our samples, *E. splendens* rhizosphere, may explain the difference. We speculated that *E. splendens* is responsible for mitigating the effects of Cu on the rhizospheric bacterial community composition and diversity in heavily Cu-contaminated soils. Firstly, *E. splendens* decreases the concentration of extractable Cu in rhizospheric soils due to the direct uptake of roots^36^. Secondly, *E. splendens* may reduce the free Cu^2+^ activity via bonding Cu with the higher dissolved organic carbon (DOC) in the rhizosphere^37^, consequently reducing Cu toxicity. In addition to our study, it is also revealed by other studies that plants exhibit the ability to harbor bacterial composition and diversity in metals contaminated soils^25^. *Sedum alfredii*, a Zn/Cd-hyperaccumulator native to China, possesses more bacteria, actinomycetes and fungi in its rhizosphere compared with bulk soils^25^. Besides, it is noted that the low pH does not improve the extractable Cu concentration in our study, which may also be explained by the rhizospheric effects of *E. splendens*. Beyond that, horizontal gene transfer and local adaptation may also explain the little influence of Cu on the rhizospheric bacterial community composition and diversity in this study. The change of bacterial community and loss of bacterial diversity might be compensated by the horizontal gene transfer in metal resistance^38^, especially when long-term metal-contamination provides bacteria more time and spatial opportunities for local adaptation to the metal stress^39^. *Proteobacteria, Actinobacteria* and *Chloroflexi,* with strong resistance to metals in different studies^40^,^41^, were the three most abundant phyla (>10%) in this study, further supporting our hypothesis. Additionally, bacteria can release exopolymers into soils, which are strongly bound to the metals and decrease their toxicity, especially Cu^42^. All the above reasons result in the tiny change of bacterial diversity in *E. splendens* rhizosphere and the weak influence on bacterial community composition of heavy Cu contamination. However, Cu still maintains its toxicity on bacteria with active carbohydrate and lipid metabolism by lipid peroxidation and inhibiting the carbohydrate metabolism enzymes^43^, which is presented in the weak part of bacteria under Cu stress.

Soil pH, which is observed to be a key factor for the construction of soil bacterial communities, affects the phyla abundance (Fig. 2c,j), bacterial community profiles, diversity and metagenomic content in *E. splendens* rhizosphere. This is a foreseeable result and other studies hold the same opinion^44^,^45^,^46^. Soil pH can directly change the physiological status of indigenous bacteria, alter their ecological niches, and reduce the abundance of individual species that are difficult to survive in soil with unsuitable pH^44^. Besides, pH also indirectly influences bacterial community by regulating soil nutrient bioavailability, affecting the TOC/TN ratio of soil organic matters, altering nitrification efficiency, changing plant primary productivity, and impacting the mobility and sorption of metals^47^,^48^,^49^. These features explain the reason why pH is such powerful in affecting bacterial community in *E. splendens* rhizosphere.

## Methods

### Soil sample collection

Twenty-one *E. splendens* rhizospheric soil samples with different Cu concentrations were collected from three Chinese Cu mines located in Tongling city in Anhui Province (TL) (30°54′1″N, 117°49′15″E), Zhuji city in Zhejiang Province (ZJ) (29°43′23″N, 119°59′9″E), and Nanjing city in Jiangsu Province (NJ) (32°4′24″N, 119°5′33″E) in November, 2013 (Supplementary Figure S3). The Cu-accumulation ability of *E. splendens* in these three provinces had been previously reported^50^,^51^,^52^. There were seven samples in each province. We collected rhizospheric soils adhering to the roots by vigorous shaking and combined duplicates from four adjacent plants as one sample. The ice packs were then used for the 2-day transport of samples from field to laboratory. After removing small gravels and roots, soil samples (about 50~100 g per sample) were sieved through a 2-mm mesh, blended and kept in a refrigerator. Soil samples used for the analysis of physicochemical properties were kept at 4 °C, and the remaining soils for DNA extraction were stored at −20 °C.

### Physicochemical analysis

Total organic carbon (TOC) and total nitrogen (TN) were determined following Hedges’ method^53^ with some modifications. Briefly, the 1.0 g of soil sample was freeze-dried, followed by blending and treating twice with 30 mL of organic-free 1 M HCl in a 50 mL centrifuge tube. Next, the soil was washed with ultrapure water to a final pH value of 6–7 and freeze-dried again. TOC and TN were then analyzed with an elemental analyzer (vario EL cube, Elementar Analysensysteme GmbH). Soil total Cu, composed of all types of Cu including the fractions hardly absorbed by bacteria or plants, was determined using the flame-atomic absorption spectrometer (AAS; novAA 400, Analytik Jena AG) after homogenization and strong acid digestion (4:1 concentrated HNO_3_ and HClO_4_, v/v) for 32 h. The CaCl_2_ extractable Cu, representing the bioavailable Cu, was determined by flame-AAS after shaking 2.5 g of soil with 12.5 mL of a 0.01 M CaCl_2_ solution for 2 h. The standard sample (GBW07410) was measured to estimate the recovery efficiency and the recovery was 94.5% for Cu. Soil pH was measured in a suspension with 1:5 (w/v) soil/0.01 M CaCl_2_ solution using a pH meter^3^.

### DNA extraction

Soil DNA was extracted from 0.5 g of the homogeneous soil using a PowerSoil DNA Isolation Kit (MO BIO Laboratories) with a final elution in 50 *μ*L deionized water according to the manufacturer’s instruction. Triple times extraction was performed per sample. Then, the DNA solutions were combined and the concentration/quality was measured using a NanoDrop 2000 Spectrophotometer (NanoDrop Technologies). The DNA sample was stored at −20 °C for further analysis.

### Amplification and bar-coded pyrosequencing of bacterial 16S rRNA genes

The V4 hypervariable region (~300 bp) was amplified to provide sufficient resolution for accurately classifying the taxonomy of bacterial sequences. Polymerase chain reaction (PCR) was performed using the universal primer (515F 5′-GTGCCAGCMGCCGCGGTAA-3′ and 806R 5′-GGACTACHVGGGTWTCTAAT-3′) for nearly all-bacterial taxa^54^. A unique 12-bp barcode was incorporated into the 806R primer to distinguish the amplified products. The link code and pad sequence are located between the barcode and primer. The sample IDs, primers, and their congruent relationships are shown in Supplementary Table S4. PCR mixtures (50 *μ*L) contained the following components: 50–100 ng (1 *μ*L) of template DNA, 25 *μ*L of rTaq premix buffer (TaKaRa), 100 nM of each primer (1 *μ*L), and 22 *μ*L ultrapure water. The amplification in duplicates followed the procedure: 94 °C for 5 min; 28 cycles at 94 °C for 30 s, 55 °C for 30 s, 72 °C for 90 s; and a final extension at 72 °C for 5 min. Duplicate PCR products for each sample were pooled and purified using the MicroElute Cycle-Pure Kit (Omega Bio-Tek) following the manufacturer’s instruction. Then, the purified PCR products for pyrosequencing were quantified as previously and combined with approximately equimolar amounts. Before sequencing, the DNA length was detected by agarose gel electrophoresis, and the DNA concentration and quality were confirmed using a Qubit fluorometer (Thermo Fisher Scientific). Sequencing was then performed at Macrogen (Seoul, South Korea) using the Miseq PE200 sequencer.

### Processing of pyrosequencing data

The raw reads have been deposited in the National Center for Biotechnology Information (NCBI, BioProject ID: PRJNA306969, Accession number: SAMN04370756~SAMN04370776). Raw data were processed and analyzed according to Mothur^55^ and Quantitative Insights Into Microbial Ecology (QIIME)^56^. Briefly, low-quality reads with a quality score <25 or a length <250 bp were removed. Sequences were assembled based on overlap and assigned to samples according to their unique barcodes. Then, the singletons were removed which reduce the error rate with a small reduction in sensitivity. Operational taxonomic units (OTUs) with 97% similarity were picked out using Uclust^57^. Next, the representative sequence set was picked and aligned, and the chimeric sequences identified by the UCHIME algorithm^58^ were then discarded. The taxonomic classification of phylotypes was assigned to the Greengenes 13.5 database using “assign_taxonomy.py” in QIIME with default sets. The relative abundance of each taxon within each community was determined by comparing the number of sequences with the total sequences obtained from each sample.

To compare the bacterial diversity at the same level, a subset of 50,000 sequences per sample was randomly selected to normalize the sequences. The community α-diversity was determined using Chao1, Shannon and Simpson indices. The rarefaction curves were plotted based on the observed OTUs. The β-diversity characterized by UniFrac in this study, measuring the phylogenetic distance between samples, was used to determine whether the communities differ significantly. Here, weighted UniFrac matrices were calculated using QIIME scripts.

### Predictive functional profiling of bacterial communities using 16S rRNA gene

The prediction analysis was performed using PICRUSt software package. Briefly, the closed-reference OTU was picked against the Greengenes database (13.5). To reflect the accuracy abundance of gene, the OTU number was normalized by “normalize_by_copy_number.py” script. Then the metagenomes were predicted against KEGG database and the accuracy of metagenome predictions were estimated by the Nearest Sequenced Taxon Index (NSTI)^28^. KEGG has been organized into 4 levels of hierarchies. The level one is the most general categories and the level four is the most specific in each KO terms. The analyses based on PICRUSt prediction were conducted on the level 2 after grouping the predicted KOs into a higher level of categorization.

### Statistical analysis

Correlations between bacteria communities and environmental factors were determined using the taxonomy-supervised analysis (phyla abundance as variables), which is more tolerant of sequencing errors and often used to analyze the bacterial community attributing to no requirement for exhaustive computation on the alignment and clustering^59^. Eleven dominant phyla (>1%) of bacteria, pH, TOC, TN, TOC/TN, total Cu and extractable Cu were analyzed using Pearson’s correlations (two-tail) running on SPSS to examine the relationship between the relative abundance of dominant phyla and environmental factors. SPSS was also used for calculating the relationship between α-diversity and environmental factors. Additionally, the relationship between gene families abundance and environmental factors was executed using the same method described above to understand the influential factors of gene abundance. CCA and the permutation test were performed using “vegan” package based on R. MRT constructed using the R package “mvpart” with default sets was applied to understand the correlation between the relative abundance of the phyla and the environmental factors^60^. The Mantel test, a versatile statistical test that was checked by the “compare_distance_matrices.py” in QIIME package with two-tailed test and 999 times permutations, was used to describe the relationship between environmental factors and phylogenetic distance.

## Additional Information

**How to cite this article**: Jiang, L. *et al*. Exploring the Influence of Environmental Factors on Bacterial Communities within the Rhizosphere of the Cu-tolerant plant, *Elsholtzia splendens. Sci. Rep.* **6**, 36302; doi: 10.1038/srep36302 (2016).

**Publisher’s note**: Springer Nature remains neutral with regard to jurisdictional claims in published maps and institutional affiliations.

## Supplementary Material

Supplementary Information

## Figures and Tables

**Table 1:** The α-diversity indices across all samples.

	Chao1	Shannon	Simpson	Observed species
NJ1	12175	10.52	1.00	8606
NJ2	11875	10.57	0.99	9144
NJ3	10912	9.99	0.99	7429
NJ4	12018	10.20	0.99	8988
NJ5	11352	10.72	1.00	9103
NJ6	10270	9.26	0.99	6673
NJ7	10860	9.92	0.99	8554
TL1	9575	9.90	0.99	7072
TL2	9922	9.86	0.99	7027
TL3	9198	8.79	0.97	7282
TL4	11103	9.82	1.00	7285
TL5	11389	10.00	1.00	8186
TL6	9283	9.59	0.99	6264
TL7	9407	10.02	0.99	6404
ZJ1	9254	9.41	0.99	6560
ZJ2	10801	10.16	1.00	7497
ZJ3	11204	10.28	1.00	8107
ZJ4	12209	10.45	1.00	8966
ZJ5	9149	9.96	0.99	6697
ZJ6	11307	10.57	1.00	8093
ZJ7	8562	9.96	0.99	5792

**Table 2:** The average phyla abundance of each node in MRT.

Split condition	Split 1		Split 2	
	pH> = 5.75	pH < 5.75	TOC/TN> = 7.1	TOC/TN < 7.1
*Proteobacteria*	0.28	0.21	0.29	0.27
*Actinobacteria*	0.14	0.12	0.17	0.12
*Chloroflexi*	0.09	0.16	0.11	0.07
*Verrucomicrobia*	0.10	0.10	0.07	0.12
*Acidobacteria*	0.10	0.08	0.09	0.10
*Bacteroidetes*	0.07	0.04	0.07	0.07
*Planctomycetes*	0.04	0.05	0.04	0.04
*Cyanobacteria*	0.04	0.03	0.03	0.04
*Firmicutes*	0.04	0.03	0.03	0.05
*Gemmatimonadetes*	0.02	0.01	0.03	0.02
*TM7*	0.02	0.01	0.02	0.02
*Other*	0.06	0.16	0.05	0.07
