# Supplementary material for: Exploring the Influence of Environmental Factors on Bacterial Communities within the Rhizosphere of the Cu-tolerant plant, Elsholtzia splendens
Source: Sci Rep. 2016 Oct 26;6:36302. doi: 10.1038/srep36302 (PMC5080579; doi:10.1038/srep36302)
Supplement: Supplementary Information [file srep36302-s1.pdf]

Supplementary Information

Exploring the Influence of Environmental Factors on Bacterial Communities within the Rhizosphere of the Cu-tolerant plant, *Elsholtzia splendens*

Longfei Jiang[a, b], Mengke Song[b], Li Yang[a], Dayi Zhang[c], Yingtao Sun[b], Zhenguo Shen[a,]*, Chunling Luo[b,]*, Gan Zhang[b]

[a] *College of Life Sciences, Nanjing Agricultural University, Nanjing 210095, China*

[b] *Guangzhou Institute of Geochemistry, Chinese Academy of Sciences, Guangzhou 510640, China*

[c] *Lancaster university, Lancaster Environment Centre, Lancaster, LA1 4YW, UK*

* Corresponding authors:

Prof. Zhenguo Shen, E-mail: zgshen@njau.edu.cn; Tel.: +86-25-84396391; Fax:+86-25-84396325

Prof. Chunling Luo, E-mail: clluo@gig.ac.cn; Tel.: +86-20-85290290; Fax: +86-20-85290706

Table S1: Soil physicochemical characteristics (Mean±SD)

Sample	Total Cu (mg/kg)	Extractable Cu (mg/kg)	TN (‰)	TOC (‰)	TOC/TN	pH
NJ1	1770±151	14.3±0.67	0.262±0.0001	1.78±0.040	6.81	6.2±0.22
NJ2	2090±283	61.4±3.17	0.292±0.001	2.06±0.144	7.05	6.1±0.08
NJ3	2470±89	36.4±2.55	0.408±0.019	2.97±0.046	7.27	6.4±0.14
NJ4	728±24	12.5±2.64	0.23±0.022	1.33±0.058	5.81	5.8±0.14
NJ5	3050±52	52.3±0.30	0.257±0.021	1.41±0.053	5.48	6.8±0.14
NJ6	2470±119	92.1±1.03	0.324±0.008	2.37±0.065	7.32	6.2±0.08
NJ7	1190±142	8.34±0.11	0.157±0.002	0.55±0.001	3.54	6.5±0.08
ZJ1	1280±85	14.4±0.11	0.704±0.003	6.00±0.009	8.53	3.7±0.14
ZJ2	1890±174	5.19±0.13	0.464±0.101	2.49±0.034	5.37	3.7±0.081
ZJ3	3000±278	31.8±0.25	0.251±0.006	1.79±0.033	7.15	6.7±0.21
ZJ4	2900±73	25.9±2.7	0.385±0.006	2.21±0.106	5.73	6.6±0.08
ZJ5	1470±342	10.9±0.26	0.563±0.012	3.37±0.032	5.99	5.7±0.08
ZJ6	2550±179	28.3±0.27	0.833±0.013	7.62±0.173	9.15	6.2±0.08
ZJ7	4180±185	86.8±3.94	0.394±0.006	4.45±0.041	11.3	3.8±0.14
TL1	1760±142	22.1±0.07	0.602±0.017	4.05±0.018	6.72	6.3±0.14
TL2	4260±236	9.51±0.18	0.593±0.008	3.87±0.041	6.52	5.8±0.08
TL3	3290±361	29.5±0.46	0.525±0.019	3.29±0.079	6.27	6.0±0.14
TL4	3730±357	25.2±0.51	0.398±0.082	5.76±0.045	14.5	6.6±0.08
TL5	2240±56	21.5±0.34	1.173±0.068	8.80±0.130	7.50	6.5±0.14
TL6	2860±135	21.9±2.09	0.735±0.0330	5.54±0.179	7.53	6.4±0.08
TL7	5300±430	55.3±1.20	0.477±0.084	4.13±0.087	8.65	6.7±0.08

Table S2: The relationship between microbial diversity and environmental factors. The numeric value out of the bracket is *r*. The *p* value was showed in the bracket.

	Chao1	Shannon	Simpson	Observed species
Total Cu	-0.326 (0.150)	-0.088 (0.704)	0.007 (0.976)	-0.403 (0.070)
Extractable Cu	-0.179 (0.438)	-0.051 (0.827)	0.012 (0.959)	-0.230 (0.316)
TN	-0.281 (0.217)	-0.221 (0.335)	0.039 (0.866)	-0.325 (0.151)
TOC	-0.282 (0.215)	-0.199 (0.386)	0.126 (0.585)	-0.407 (0.067)
TOC/TN	-0.205 (0.372)	-0.115 (0.620)	0.215 (0.350)	-0.439 (0.046)*
pH	0.413 (0.063)	0.209 (0.364)	0.024 (0.919)	0.416 (0.060)

*represent the significant relationship

Table S3: The Nearest Sequenced Taxon Index (NSTI) value.

Sample ID	NSTI
NJ1	0.17
NJ2	0.18
NJ3	0.19
NJ4	0.16
NJ5	0.19
NJ6	0.15
NJ7	0.19
TL1	0.18
TL2	0.18
TL3	0.18
TL4	0.17
TL5	0.17
TL6	0.17
TL7	0.18
ZJ1	0.18
ZJ2	0.18
ZJ3	0.18
ZJ4	0.19
ZJ5	0.19
ZJ6	0.18
ZJ7	0.17

Table S4: The sample ID (TL: Tongling city, ZJ: Zhuji city, NJ: Nanjing city), primer and their congruent relationship

Sample	Primer (barcode+pad+linker+special primer)
	515F-TATGGTAATTGTGTGCCAGCMGCCGCGGTAA
NJ1	806rcbc60-CGGTCAATTGACAGTCAGTCAGCCGGACTACHVGGGTWTCTAAT
NJ2	806rcbc61-GTGGAGTCTCATAGTCAGTCAGCCGGACTACHVGGGTWTCTAAT
NJ3	806rcbc62-GCTCGAAGATTCAGTCAGTCAGCCGGACTACHVGGGTWTCTAAT
NJ4	806rcbc63-AGGCTTACGTGTAGTCAGTCAGCCGGACTACHVGGGTWTCTAAT
NJ5	806rcbc64-TCTCTACCACTCAGTCAGTCAGCCGGACTACHVGGGTWTCTAAT
NJ6	806rcbc65-ACTTCCAACTTCAGTCAGTCAGCCGGACTACHVGGGTWTCTAAT
NJ7	806rcbc66-CTCACCTAGGAAAGTCAGTCAGCCGGACTACHVGGGTWTCTAAT
ZJ1	806rcbc67-GTGTTGTCGTGCAGTCAGTCAGCCGGACTACHVGGGTWTCTAAT
ZJ2	806rcbc68-CCACAGATCGATAGTCAGTCAGCCGGACTACHVGGGTWTCTAAT
ZJ3	806rcbc69-TATCGACACAAGAGTCAGTCAGCCGGACTACHVGGGTWTCTAAT
ZJ4	806rcbc70-GATTCCGGCTCAAGTCAGTCAGCCGGACTACHVGGGTWTCTAAT
ZJ5	806rcbc71-CGTAATTGCCGCAGTCAGTCAGCCGGACTACHVGGGTWTCTAAT
ZJ6	806rcbc72-GGTGACTAGTTCAGTCAGTCAGCCGGACTACHVGGGTWTCTAAT
ZJ7	806rcbc73-ATGGGTTCCGTCAGTCAGTCAGCCGGACTACHVGGGTWTCTAAT
TL1	806rcbc74-TAGGCATGCTTGAGTCAGTCAGCCGGACTACHVGGGTWTCTAAT
TL2	806rcbc75-AACTAGTTCAGGAGTCAGTCAGCCGGACTACHVGGGTWTCTAAT
TL3	806rcbc76ATTCTGCCGAAGAGTCAGTCAGCCGGACTACHVGGGTWTCTAAT
TL4	806rcbc77-AGCATGTCCCGTAGTCAGTCAGCCGGACTACHVGGGTWTCTAAT
TL5	806rcbc78-GTACGATATGACAGTCAGTCAGCCGGACTACHVGGGTWTCTAAT
TL6	806rcbc79-GTGGTGGTTTCCAGTCAGTCAGCCGGACTACHVGGGTWTCTAAT
TL7	806rcbc80-TAGTATGCGCAAAGTCAGTCAGCCGGACTACHVGGGTWTCTAAT

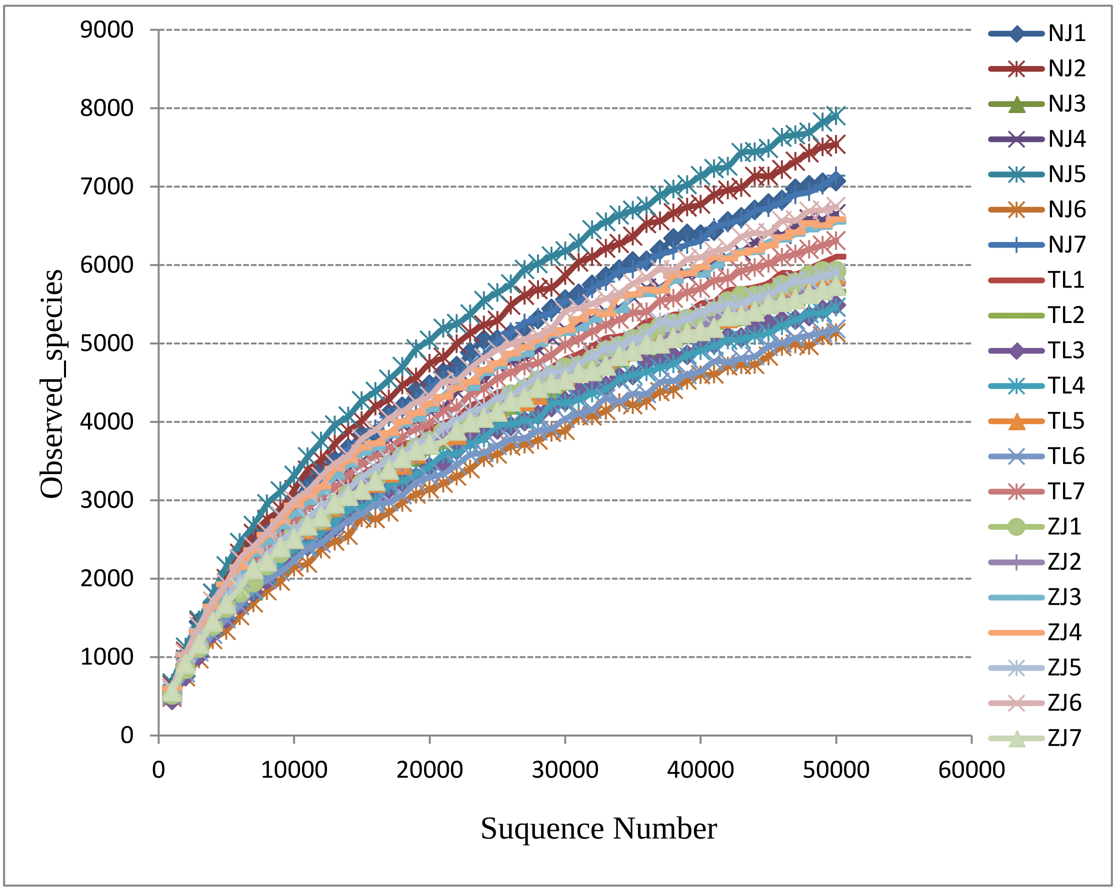

Figure S1: Rarefaction curve based on observed OTUs.

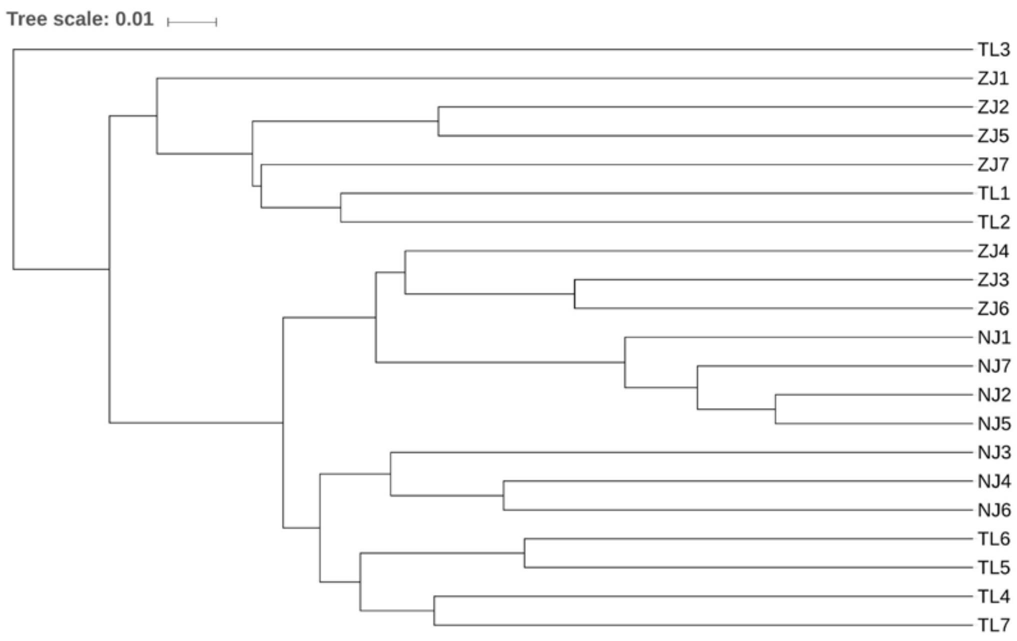

Figure S2: Unifrac distance between different samples.

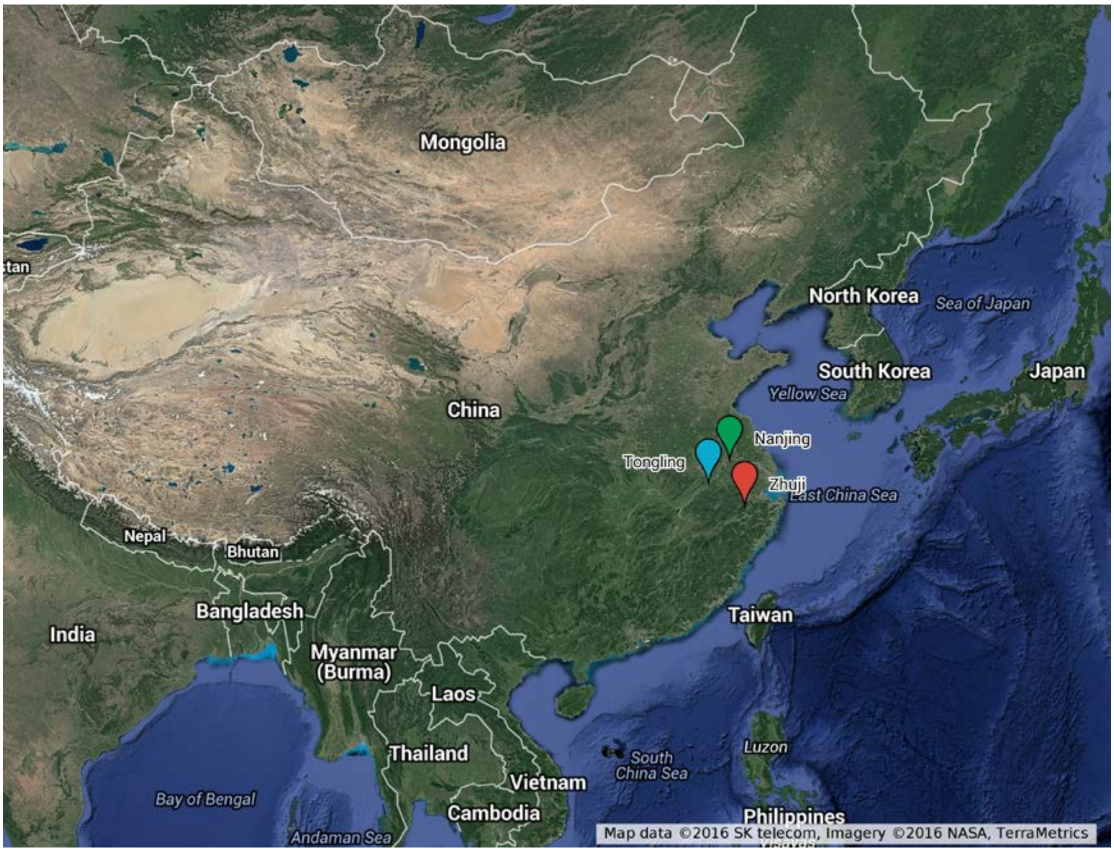

Figure S3: Sampling sites (Data from google map: Map data © 2016 SK telecom, Imagery © 2016 NASA, TerraMetrics).
